# Biosensor applications in organ-on-a-chip platforms and disease modeling

**DOI:** 10.3389/fbioe.2026.1824674

**Published:** 2026-07-21

**Authors:** Elizabeth Coln, Chase Miller, James J. Hickman, Christopher J. Long

**Affiliations:** 1 Hesperos, Inc., Orlando, FL, United States; 2 Nanoscience Technology Center, University of Central Florida, Orlando, FL, United States

**Keywords:** biosensors, blood-brain barrier (BBB), disease modeling, heart-on-a-chip, microelectrode arrays (MEA), microphysiological system (MPS), organ-on-a-chip (OOC), trans-epithelial/endothelial electrical resistance (TEER)

## Abstract

Research efforts are advancing with the use of microphysiological systems (MPS) and organ-on-a-chip systems (OoCs) integrated with biosensors for *in vitro* modeling of cell and tissue function for a better understanding of dynamic cellular microenvironments and disease mechanisms. OoC systems coupled with biosensors enable more accurate modeling of human physiology and disease. These platforms provide non-invasive, enhanced real-time monitoring of cellular behaviors and responses with high sensitivity and selectivity and allow for more physiologically and pharmacologically relevant models for high-throughput drug screening, systemic disease modeling, pharmacokinetic modeling, and personalized medicine. The integration of biosensors allows for precise detection of biomarkers, physiological changes, and drug effects, providing valuable insights into disease mechanisms, drug toxicity, and therapeutic efficacy. This review provides an overview of biosensor types and recent advancements with particular focus on applications to *in vitro* modeling of cell and tissue function. Specific examples of organ-on-a-chip systems integrated with biosensors are then highlighted, including applications in disease modeling.

## 1 Introduction

Microphysiological systems (MPS) and organ-on-a-chip (OoC) models are microfluidic devices engineered with both 2D and 3D cellular models of tissue constructs used to simulate *in vivo* human organs and organ systems (Liu et al., 2023; Dey et al., 2024). Combinations of organ models in multi-organ and body-on-a-chip (BoaC) systems enable advanced investigations to simulate the complex interactions across multiple organs in the body. The increased complexity of multi-organ models and BoaC platforms allow for high-throughput drug screening, systemic disease modeling, pharmacokinetic modeling, and personalized medicine with improved physiological and pharmacological relevance compared to more standard conventional *in vitro* culture counter parts (Kim et al., 2023; Liu et al., 2023).

Biosensors are often integrated into MPS and OoC systems to enable in-line and non-invasive monitoring of specific functional readouts from cellular constructs and provide rapid, real-time detection and efficient screening (Busek et al., 2022; Kim et al., 2023). Biosensors can be described as devices designed to monitor biological systems and cellular environments, providing information through measurable outputs. By using chemical, biological, and physical detection methods, biosensors offer an effective approach for real-time, *in situ* monitoring of microphysiological systems (Akcay et al., 2024). Zhu et al. reviewed the variety of sensing modalities seen in modern OoC devices, ranging from direct measurement of electrical and mechanical output of cellular action to various methods of quantification and characterization of biochemical indicators of cellular activity (Zhu et al., 2021). Commonly used biosensors include electrochemical sensors used to monitor parameters such as pH, oxygen, and glucose which can be integrated into OoC systems for real-time observation of cellular interactions (Liu et al., 2023; Huang et al., 2024). Other common applications of electrochemical biosensors in MPS include transepithelial/endothelial electrical resistance (TEER) measurements to monitor integrity and permeability of blood-brain barrier (BBB) models, detection of biomarkers associated with hepatotoxicity and liver model injury induced by drug compounds, and investigation of metabolic mechanisms in kidney models (Öztatlı et al., 2023). Precise measurement of cellular microenvironments may additionally be performed by various optical sensors with many target analytes to indicate cellular activity and response to stimuli, such as oxidation levels driven by cellular respiration as well as detection of changes in dissolved oxygen as it is consumed (Yang W. et al., 2024). Many types of sensors have been developed for MPS to measure cellular action, such as pillar arrays, field-effect transistors (FETs), and microelectrode arrays (MEAs). MEAs are of particular interest in recent advances and have been used extensively for electrophysiological analysis of electrically active cellular constructs. Applications involving neuronal cell types commonly employ MEAs, such as the human-based functional nociceptor MEA platform developed by Nimbalkar *et al.* as a pain model for the evaluation of effective analgesics and drug development (Nimbalkar et al., 2023).

**FIGURE 1:**
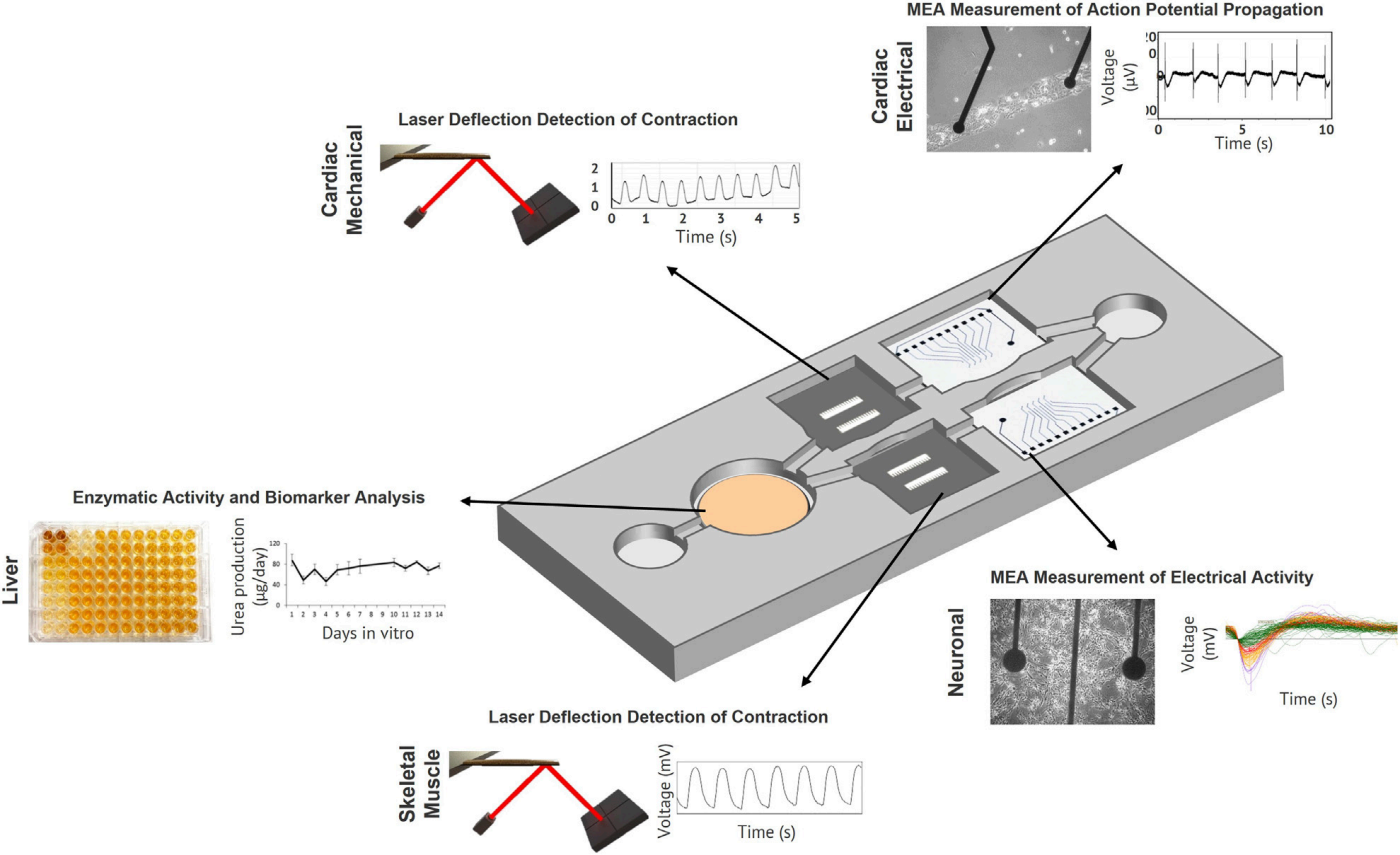
Example of a multi-organ microphysiological system (MPS) with integrated biosensors. Adapted with permission from “Microphysiological system to address the opioid crisis: A novel multi-organ model of acute opioid overdose and recovery” by Patel et al., published in *Current Research in Toxicology*, *8*, 100,209 (2025). Copyright © 2025 Elsevier. All rights reserved.

Combinations of OoC models in multi-organ MPS often benefit from the integration of multiple biosensor types such as the multi-organ system shown in Figure 1 (Patel et al., 2025). Patel *et al.* demonstrated a multi-organ MPS consisting of cardiac, skeletal muscle, preBötzinger complex (preBötC) neurons, and hepatocytes to model acute opioid overdose and recovery. This multi-organ platform enabled investigation of functional effects of the opioid methadone and rescue agent naloxone using cantilever-based sensing and MEAs for contractile muscle force and electrical activity measurements, respectively (Patel et al., 2025). This review describes the different types of biosensors used for *in vitro* modeling of cell and tissue function with a focus on recent advancements and applications within OoC systems and disease modeling.

## 2 Types of biosensors

Biosensors can typically be classified as optical, electrochemical (label-based or label free), electrical (conductometric) and physical based on method of signal transduction (Paul et al., 2024). Optical and electrochemical biosensors for MPS are typically the most simplistic and cost-effective and can be miniaturized for integration with microfluidic MPS, which are commonly used in drug screening, toxicology, and disease modeling (Mou et al., 2022; Hussain Memon et al., 2023). From a traditional perspective, biosensors have been defined as a sensor requiring an isolated biorecognition element, such as enzymes or antibodies (Thevenot et al., 2001). However, within MPS, this definition is often expanded (Kim et al., 2023; Mou et al., 2024). Because the cellular constructs themselves function as the biological recognition components within OoC platforms, we adopt this broader paradigm. This allows for the inclusion of essential electrical and physical approaches, such as MEAs, cantilevers, and TEER sensors, that directly interface with and monitor dynamic biological functions in MPS. This microphysiological framework aligns with emerging consensus classifications that categorize on-chip architectures into unified electrical, optical, and mechanical sensing modalities to achieve continuous evaluation (Ling et al., 2026). Here we review several common biosensor types and applications to functional measurement of cells and tissue function. While the fundamental physics, material fabrications, and surface chemistries of these transduction mechanisms are reviewed in extensive detail elsewhere (Zhu et al., 2021; Mou et al., 2022; Öztatlı et al., 2023) the following sections outline their baseline operating principles as they apply to integrated on-chip monitoring within this expanded microphysiological framework.

### 2.1 Optical biosensors

Optical biosensors detect changes in light properties when interacting with target biomarkers (Zhang et al., 2024). These systems generally transduce information by fluorescence, absorbance, reflectance, and luminescence, with fluorescence and chemiluminescence being two of the most widely used techniques (Dehghandehnavi et al., 2024; Paul et al., 2024). Other common techniques for optical detection of biomarkers include colorimetry and interferometric measurements as well as surface plasmon resonance (SPR), which is a label-free method that enables real-time monitoring of biomolecular interactions such as protein and nucleic acid dynamics (Mou et al., 2022; Paul et al., 2024; Zhang et al., 2024). By eliminating the need for direct electrical wire connections, these sensors are frequently integrated into OoC platforms to enable the selective and sensitive real-time detection of analyte such as pH, glucose, and oxygen (Fedi et al., 2022; Mou et al., 2022; Hussain Memon et al., 2023; Paul et al., 2024; Zhang et al., 2024). Removing invasive electrical components that can disrupt delicate tissues provides a means to capture real-time metabolic shifts, dissolved oxygen depletion, and biomarker variations directly within the microfluidic environment.

### 2.2 Electrochemical biosensors

Electrochemical biosensors combine a biorecognition element with a transducer to convert interactions with a target biomarker into an electrical signal. The corresponding electrical signal is correlated to the concentration of the specific analyte being detected, often using a calibration of standard solutions. Electrochemical detection methods include voltammetry/amperometry, electrochemical impedance spectroscopy, conductometry, and potentiometry (Fedi et al., 2022; Hussain Memon et al., 2023; Paul et al., 2024). A common application of electrochemical biosensors is in the study of cellular metabolism, such as the light-addressable potentiometric sensor (LAPS)-based platform developed by Tian et al., for long-term monitoring of extracellular calcium metabolism for *in vitro* drug evaluation (Tian et al., 2025). Various types of electrochemical biosensors (e.g., pH, glucose, oxygen) have been used with 3D hydrogel-based tissue models to monitor cellular metabolism as well (Fedi et al., 2022). Electrochemical sensors are often paired with other types of sensors, such as the Cu^2+^ and Zn^2+^ ion selective permeability measurement system described by Sciurti et al., which served as a compliment to TEER measurements in their transwell tissue models (Sciurti et al., 2023). While enzyme or reactant lifetime is a key factor limiting the longevity of electrochemical biosensors for continuous use applications, due to their high sensitivity and specificity, these biosensors are often incorporated into OoC systems for biomarker monitoring (Hussain Memon et al., 2023; Paul et al., 2024; Zhang et al., 2024). By converting biochemical interactions directly into electrical outputs, electrochemical biosensors provide a reliable method to monitor cell viability and real-time metabolic shifts during long-term drug screening applications.

### 2.3 Electrical biosensors

Electrical biosensors detect changes in voltage or current to indicate biological activity. For example, integrated electrode pairs in impedance-based sensors are used in OoC systems to measure TEER, a common method of assessing barrier integrity in blood-brain barrier (BBB) and gut models (Mou et al., 2022). Biosensors for barrier integrity measurements have been the subject of many investigations regarding their fabrication, throughput, and sensitivity with respect to the multitude of options for integration within MPS (Arman et al., 2024; Ugodnikov et al., 2024). One such example is demonstrated by Zhu et al. in the development of an *in vitro* external field-effect transistor (FET) incorporated with a flexible PDMS biosensor. This technique allowed for real-time barrier integrity monitoring of a synthetic lipid bilayer membrane to study the interactions between a cell membrane model and traversing nanoparticles (Zhu et al., 2025). A more common application of electrical biosensors is the use of microelectrode arrays (MEAs), which measure extracellular local field potentials, offering a less invasive and higher throughput alternative to techniques such as patch clamp. Improvements to microfabrication techniques have allowed for the creation of intensified MEAs such as the nanowell patterned MEA developed by Xiang et al. which enabled measurement of the intracellular action potential of a single cardiomyocyte (Xiang et al., 2022). Additionally, high-density MEAs (HD-MEA) have been developed for feats such as measuring dynamic spatial variations in impedance of epithelial barrier tissue to monitor barrier cell proliferation and differentiation (Venz et al., 2025). In general, electrical biosensors, such as those used for TEER measurements and MEAs, are essential tools for *in vitro* modeling and drug testing as they evaluate cellular function and tissue health by directly tracking impedance drops in degrading cellular barriers or electrophysiological variations like field potential duration (FPD).

### 2.4 Physical biosensors

Physical biosensors detect measurable quantities through effects such as force, heat, light, and electrical signals. Examples include microelectromechanical systems (MEMS) resonant mass sensors, cantilever-based sensors, and piezoelectric sensors (Paul et al., 2024; Saikia et al., 2025). Of particular interest are mechanical sensors used for the evaluation of stress, strain, change in mass of biomolecules, and force. Piezoelectric materials have been widely used in biomedical applications and OoC systems for monitoring mechanical force from tissues due to their high specificity, sensitivity and real-time measurements (Zhang et al., 2024; Zhang et al., 2025). Advancements in nanotechnology have enabled further development of silicon-based microcantilever biosensors using complementary metal–oxide–semiconductor (CMOS)-compatible microfabrication techniques. These mechanical sensors detect changes in resonance frequency or mechanical deflections resulting from biomolecular interactions on the microcantilever’s surface. Microcantilever biosensors are well-known for their enhanced sensitivity, potential for miniaturization, and compatibility with advanced electronics (Muhammad et al., 2025). An example of the application of microcantilever biosensors in MPS is demonstrated by Jangir et al., who developed a functional platform that replicated the tendon extracellular matrix (ECM) using silicon microcantilevers. This platform allowed for an investigation into the force dynamics in support of the long-term *in vitro* survival of mechanically active skeletal muscle myotubes for integration into microphysiological platforms (Jangir and Hickman, 2023). These physical biosensors prove vital for translating dynamic mechanical actions, such as the beating of a cardiac syncytium or the contractile force of a skeletal muscle model, into raw biomechanical data to characterize tissue development and evaluate drug efficacy.

## 3 Organ-on-a-chip applications

Biosensor integration into OoC platforms is transforming the field of biomedical research by enabling real-time, non-invasive monitoring of cellular and tissue responses in a controlled microenvironment. These biosensors allow for precise detection of biomarkers, physiological changes, and drug effects, providing valuable insights into disease mechanisms, drug toxicity, and therapeutic efficacy. Here we review recent applications of biosensors integrated with various OoC models for monitoring cell and tissue function, with examples presented in Table 1.

**TABLE 1:** Biosensors in organ-on-a-chip models.

Organ type	Biosensor type	Measurement technique	Application	Measurement metric	Reference
Brain	Electrical	MEA	Electrophysiology	Neuronal activity and action potential	Gallo et al. (2024), Saglam-Metiner et al. (2024)
	​	​	​	Neural network characteristics in AD disease type models	Gallo et al. (2024), Saglam-Metiner et al. (2024)
	​	TEER	Barrier integrity	Blood-brain barrier (BBB) integrity and drug toxicity	Autar et al. (2022), Cecen et al. (2023), Gallo et al. (2024)
	Electrochemical	Voltametric and Amperometric Sensors	Environmental Analyte Detection	Ion concentrations and pH acidification	Cecen et al. (2023)
	​	​	​	Oxygen concentration and cellular respiration	Cecen et al. (2023)
	​	​	​	Glucose and lactate for cellular metabolism	Cecen et al. (2023)
	​	Enzyme-Based Sensors	Biological indicators	Reactive Oxygen Species (ROS) production for neuroinflammation indicators	Cecen et al. (2023)
	Optical	Colorimetric Sensors	Environmental Analyte Detection	pH, glucose, nicotinamide adenine dinucleotide, and 6-hydroxy dopamine	Cecen et al. (2023)
	​	​	Biomarker detection	Biomarkers of brain injury (s N-acetylasparate (NAA) and GFAP)	Cecen et al. (2023)
	​	Surface Plasmon Resonance (SPR)	Biomarker detection	Cytokine secretions	Cecen et al. (2023)
Heart	Electrical	MEA	Electrophysiology	Stimulated cardiomyocyte activity for studying arrythmias	Liu et al. (2025)
	Mechanical	Cantilever-Based Sensors	Contractability	Cardiac force output	Liu et al. (2025)
	Optical	Fluorescence-Based Sensors	Environmental Analyte Detection	Calcium detection for signal monitoring	Liu et al. (2025)
	​	​	​	Oxygen for metabolic status	Liu et al. (2025)
Lung	Optical	Photonic Ring Resonator	Biomarker Detection	Biomarkers of lung epithelial inflammation	Cognetti et al. (2023)
	​	Colorimetric Sensors	Environmental Analyte Detection	Non-invasive media-based pH measurement	Khalid et al. (2020)
	Electrical	TEER	Barrier Integrity	Toxicity of doxorubicin and docetaxel	Khalid et al. (2020)
Gut	Electrical	TEER	Barrier Integrity	Barrier formation and integrity	Giampetruzzi et al. (2022), Brandauer et al. (2025)
Liver	Electrochemical	Amperometric Sensors	Biomarker Detection	Cell secreted lactate, IL-6, and GST-α	Yang J. W. et al. (2024)
	Optical	Photonic Crystal-Total Internal Reflection (PC-TIR)	Label Free Biomarker Detection	Selective monitoring of liver-secreted albumin and GST-α	Yang J. W. et al. (2024)
	​	​	​	Monitoring doxorubicin-induced liver model damage	Yang J. W. et al. (2024)
Bone	Optical	Fluorescence-Based Sensors	Biomarker Detection	Immunostaining protein biomarkers	Zhang et al. (2023)
	​	​	​	Enzymatic detection of osteoblast and osteoclast biomarkers	Zhang et al. (2023)
	​	​	​	Lipid droplet deposition from adipose tissues	Zhang et al. (2023)
	​	​	​	Calcium imaging	Zhang et al. (2023)
Cartilage	Electrochemical	Cyclic Voltammetry	Environmental Analyte Detection	Osteoarthritis-induced nitric oxide release	Belcastro et al. (2025)
	​	​	​	Osteoarthritis-induced glucose and lactate detection with needle electrodes	Rothbauer et al. (2025)

### 3.1 Brain

**FIGURE 2:**
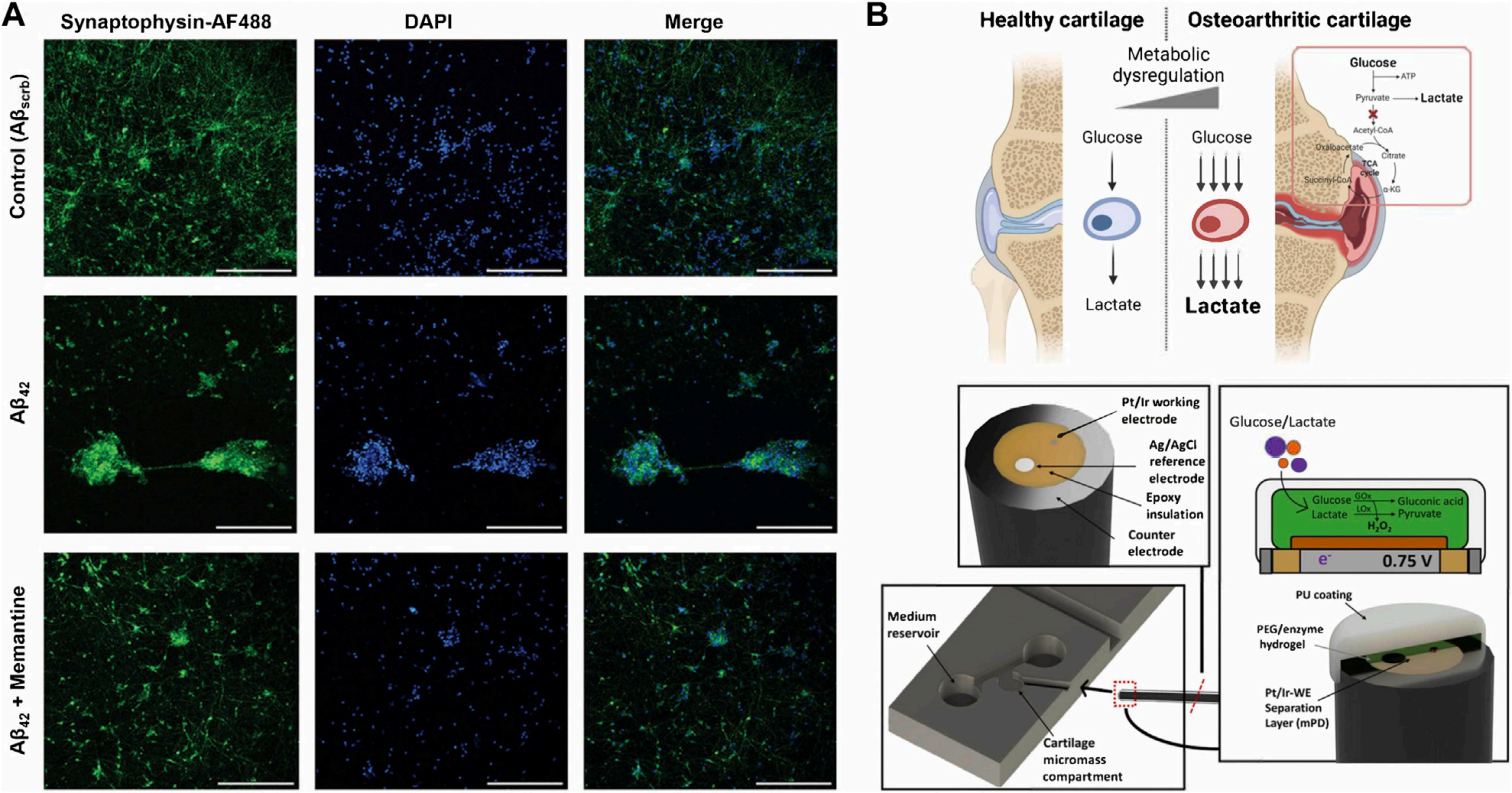
Examples of Organ-on-a-Chip disease models. **(A)** Adapted with permission from “A functional aged human iPSC-cortical neuron model recapitulates Alzheimer’s disease, senescence, and the response to therapeutics” by Gallo et al., published in *Alzheimer’s & Dementia*, *20* (9), p. 5940–5960 (2024). Copyright © 2024 John Wiley & Sons. All rights reserved. **(B)** Adapted with permission from “Integration of glucose and lactate biosensors into human cartilage-on-a-chip models for long-term monitoring of metabolic shifts in osteoarthritis” by Rothbauer et al., published in *Sensors and Actuators* B*: Chemical*, *427*, 137,123 (2025). Copyright © 2025 Elsevier. All rights reserved.

Brain-on-a-chip microfluidic platforms have been utilized to study the central nervous system (CNS) and the BBB to model diseases such as Alzheimer’s disease (AD), Parkinson’s disease (PD), and Huntington’s disease (HD) (Cecen et al., 2023). The integration of various types of biosensors, such as electrical, optical, and colorimetric sensors, allow OoCs to monitor a range of features and offers advantages for monitoring multi-organ platforms (Cecen et al., 2023). BBB-on-a-chip devices integrated with biosensors provide a noninvasive, real-time method for monitoring BBB integrity, often through TEER measurements, as well as for studying drug delivery in both healthy and diseased models (Kincses et al., 2023). MEAs are commonly used for electrophysiological measurements of neuronal activity and can detect neuronal action potentials (spikes) by measuring changes in the transmembrane or extracellular voltage of the cells (Saglam-Metiner et al., 2024). MEAs have been used for studying disease models such as the functional progerontic cortical neuron model developed by Gallo et al. that mimics major hallmarks of AD (Figure 2A), in which human-induced pluripotent stem cell (hiPSC)-derived cortical neurons were cultured on MEAs for non-invasive measurements of neuronal long-term potentiation (LTP), a cellular surrogate for learning and memory, within the living human neural network (Gallo et al., 2024). When pathogenic amyloid*-*beta 42 (A*β*_42_) was introduced, the MEAs quantitatively captured significant dampening of baseline neuronal electrical activity and a reduction in neuronal firing rates, providing a direct electrophysiological read-out of network degradation. Similarly, Autar et al. developed a phenotypic model of hiPSC-derived cortical neurons integrated with MEA technology for disease modeling of neurological disorders (Autar et al., 2022).

### 3.2 Heart and cardiovascular system

Heart-on-a-chip systems often incorporate on-chip biosensors, such as MEAs, to monitor cardiac tissue and track electrical activity (Butler and Reyes, 2024). These MEAs record key metrics such as field potential duration (FPD) and beating frequencies, allowing for the continuous evaluation of stimulated cardiomyocyte activity and potential drug-induced arrhythmias. Sensors composed of movable parts are commonly used to measure cardiac contractility by measuring displacement, through optical, microscopy, or electronic readouts. Microcantilevers, for example, can be used to measure cardiac contractility by detecting cantilever displacement caused by cardiac contractions through optical detection methods, which yields cardiac contractile force output. Additionally, to monitor tissue metabolism during ischemia-reperfusion and myocardial infarction modeling, heart-on-a-chip devices can be constructed on O_2_ sensor substrates featuring embedded O_2_ sensor spots. These sensors allow measurements of the rate of oxygen depletion during simulated ischemic events, serving as a critical metabolic endpoint which allows for a correlation between oxygen availability and anaerobic metabolism, reduced ATP production, and a decrease in myocardial contractile force (Liu et al., 2025).

Expanding from localized cardiac models to broader vascular pathologies, Lincon et al. developed a label-free sensing platform for anti-atherosclerosis drug testing. This *in vitro* system allows for the evaluation of electrical, mechanical, and chemical properties in an atherosclerosis disease model, offering a non-invasive method for studying disease progression and providing insights into the metabolic dysregulation of macrophages in an atherosclerotic environment with nanodrug treatment (Lincon et al., 2025). Criscione et al. provides a detailed review of biosensors integrated with heart-on-a-chip platforms including electrochemical, optical, piezoelectric, and magnetic biosensors and their use in modeling *in vitro* diseases such as cardiomyopathies and ischemia reperfusion injuries (Criscione et al., 2023). Heart-on-a-chip platforms integrated with biosensors allow for increased throughput, real-time assessment, and improved efficiency during pre-clinical disease modeling, drug-induced cardiotoxicity and efficacy studies, drug screening predictivity, and personalized medicine.

### 3.3 Lung and oncology models

Many recent applications of lung MPS models have been focused around oncological pursuits. Globally, tumor-on-chip (ToC) platforms have emerged as valuable tools in oncology research by providing an *in vitro* system that models the dynamic tumor microenvironment across different stages of tumor progression, serving a crucial role in tumor treatment studies. When integrated with electrochemical biosensors, these ToC systems enable more precise investigation of tumor cells and biomarkers, providing high sensitivity and selectivity, playing a key role in the discovery, development, and testing of new therapeutics (Lei et al., 2022). Applications of this work extend past the use of MPS systems as well, such as the work outlined by Lavanya et al. in their report on applications of 2D nanomaterials-based electrochemical biosensors for the detection of various biomarkers to aid in the diagnosis of various cancers, especially ovarian cancer (Lavanya et al., 2024).

As a specific microphysiological example of oncology monitoring, Khalid et al. developed a lung cancer-on-chip platform. Integrated biosensors allowed for real-time monitoring and cytotoxicity assessment of the anti-cancer drugs doxorubicin and docetaxel, using an optical pH sensor for noninvasive monitoring of the culture media and TEER impedimetric biosensors to evaluate cytotoxicity (Khalid et al., 2020).

Applications of biosensors in lung-on-a-chip models have been applied to other investigations as well, such as that performed by Cognetti et al. where incorporated photonic biosensors for inflammatory cytokines in a two-channel, microfluidic human tissue chip model, enabled label-free, real-time detection of specific lung epithelial inflammatory biomarkers. Additionally, they demonstrated the system’s ability to monitor analyte transport following disruption of the tissue barrier (Cognetti et al., 2023).

### 3.4 Gut

A polydimethylsiloxane (PDMS)-based microfluidic gut-on-a-chip model, such as the platform developed by Brandauer et al.*,* has been integrated with membrane-based electrode microarrays to monitor epithelial barrier formation and senescence-mediated changes in intestinal barrier integrity. The *in vitro* system described featured a fixed position porous membrane-based impedance sensor, offering higher sensitivity than traditional chopstick electrodes (Brandauer et al., 2025). Advancements in material composition of MPS and biosensors have enabled the development of an intestinal barrier-on-a-chip (IBoC) platform using human Caco-2 cells integrated with transparent TEER electrodes to monitor cell growth by measuring impedance spectra of the barrier tissue (Giampetruzzi et al., 2022).

### 3.5 Liver

Liver-on-a-chip models have been integrated with biosensors to detect oxygen levels, temperature, barrier integrity, and biomarkers for hepatotoxicity testing and drug screening. Additionally, they can be combined with other organ systems and mathematical modeling for studying drug metabolism (Liu et al., 2024). Application of these biosensors in hepatic models has been demonstrated by Yang et al., who integrated a 3D liver-on-a-chip platform with a label-free photon crystal-total internal reflection (PC-TIR) optical biosensor to enable rapid, continuous monitoring of secreted biomarkers associated with drug-induced liver toxicity (Yang J. W. et al., 2024).

### 3.6 Musculoskeletal system (bone, cartilage, and skeletal muscle)

Studying bone and cartilage development and disease has proven difficult to perform outside of animal models due to numerous factors such as the complexity of the structure and difficulty in extracting measurements of key properties and functionality. For instance, a cartilage-on-a-chip device integrated with a 3D flexible electrochemical sensor was developed by Qin et al. for real-time detection of nitric oxide release from chondrocytes with dynamic mechanical stimulation (Qin et al., 2024). Complementing these microfluidic approaches, Belcastro et al. outlined the construction of a simple biosensor for real-time monitoring of nitric oxide production in an optimized 2D *in vitro* cell inflammation model using bovine chondrocytes, designed to simulate chronic inflammation, such as in osteoarthritis (Belcastro et al., 2025). Another recent study by Rothbauer et al. integrated enzymatic glucose and lactate needle electrode biosensors (Figure 2B) with a human osteoarthritic cartilage-on-a-chip system for long-term, real-time monitoring of osteoarthritis-related metabolic changes and for quantifying glucose and lactate concentrations in healthy and osteoarthritic models (Rothbauer et al., 2025).

Bone-on-a-chip systems face similar limitations to cartilage-on-a-chip models; however, due to the high complexity of the bone microenvironment there have been limited reports of successful applications. Zhang et al. provides a detailed review of the progress of bone-on-a-chip platform development and emphasizes the need for embedding sensors capable of tracking specific biochemical and biophysical properties in real-time (Zhang et al., 2023). Specifically, integrated optical and fluorescence-based biosensing techniques enable the capture of essential physiological endpoints within the bone-on-a-chip platform. These include the sensing of biochemical properties by immunostaining for functional protein biomarkers, enzymatic detection of cell secretions during bone remodeling, and detection of mRNA, lipids, and calcium and phosphate ions. Biophysical properties can be assessed through the Young’s modulus of the bone matrix, cell morphology, and cell migration. Tracking these distinct endpoints demonstrates how precise sensor read-outs map the functional changes associated with bone tissue remodeling, matrix mineralization, and pathological progression.

Expanding beyond hard tissues, muscle-on-a-chip devices offer a vital method for *in vitro* disease modeling of complex muscular dystrophies. These platforms can potentially accelerate the development of new therapies by replicating dynamic muscle function and incorporating biosensors for real-time monitoring. For example, Fernández-Costa et al. discussed the integration of muscle-on-a-chip with plasmonic biosensors to capture real-time measurements of protein biomarkers and monitor functional muscle responses to simulate muscular dystrophy pathologies (Fernandez-Costa et al., 2023).

## 4 Discussion

Biosensors are powerful analytical tools for *in vitro* testing of cell and tissue functions that have continued to develop through advanced manufacturing processes and materials, with further innovations driving increased use and expanded capabilities. Furthermore, OoC systems coupled with biosensors enable more accurate modeling of human physiology and disease. These platforms provide non-invasive, enhanced real-time monitoring of cellular behaviors and responses with high sensitivity and selectivity for the evaluation of cell and tissue function. As highlighted by Ling et al., this shift from traditional, offline endpoint assays to continuous *in situ* monitoring represents a vital paradigm shift, enabling the capture of transient cellular events and complex multi-organ interactions as they unfold (Ling et al., 2026). Consequently, organ-on-a-chip devices with integrated biosensors hold great potential in drug discovery, toxicity screening, disease diagnosis, and understanding disease mechanisms, while also offering the potential to revolutionize personalized medicine and targeted therapeutic treatments.

Organ-on-a-chip platforms and biosensing innovation will continue to develop in parallel, as biosensor capabilities are expanded or adapted to fill critical needs in emerging MPS applications while innovators create new capabilities in organ-on-a-chip devices to utilize advancements in biosensor technology. The recent integration of CMOS technology for biosensors is one advancement that has the potential to dramatically improve organ-on-a-chip capabilities, such as improved sensitivity and selectivity through post-fabrication surface functionalization. This technology offers the advantage that the sensor and readout circuit are on one chip, eliminating the need for external electronic instruments. This intensification in design faces challenges such as the high cost and complexity of developing CMOS electrical and magnetic biosensors (Dehghandehnavi et al., 2024). Other modern advancements include nanomaterial-based biosensors such as electrochemical and optical biosensors that have been extensively used for the detection of neurotransmitter serotonin molecules (Chavan et al., 2025).

Maximizing the reliability, longevity, and repeatability of these integrated elements will enable more expansive use of biosensor technology and allow for standardization of common sensors within defined contexts of use (Hussain Memon et al., 2023; Reyes et al., 2024). Standardizing platforms requires evaluating a biosensor’s Limit of Quantitation (LOQ) to measure baseline activities and pathological spikes. Although literature emphasizes absolute Limits of Detection (LOD), these values shift during extended culture due to sensor drift, fluid flow rates, and surface fouling. Prioritizing stable operational LOQs that map directly to expected biological ranges is far more critical for validating long-term screening platforms than chasing minimal detection limits. Moving forward, this push toward standardization must facilitate, rather than impede, innovation within either the organ-on-a-chip devices or biosensors. A careful balance must be maintained between the rigorous standardization required for industrial, automated drug screening and the flexible environments necessary for discovery-driven research. While maximizing reproducibility via uniform, immortalized cell lines is standard practice for commercial toxicology, patient-specific disease models for personalized medicine inherently feature unique donor-to-donor variations that limit traditional biological replication (Kong et al., 2026). An optional standard for biosensor connectivity, output data formatting, and physical geometries could provide easier adaptation and integration of biosensors into OoC devices and aid in development of OoC devices. However, because of the large range of disease states to be modeled, methods of action to be studied, and unique biosensor capabilities, standardization of too many aspects of each biosensor or standardization across too many disparate biosensors risks limiting innovation both in terms of biosensors and in OoC devices. An approach that enables innovation and increased capabilities while ensuring reproducibility needed to inform clinicians and regulators is to validate biosensors and systems for specific contexts of use. In cases where standardization can be applied more easily, the range of that biosensor’s context of use will be broader, while specialized contexts of use can still be addressed.
